# Supplementary material for: Federated privacy-protected meta- and mega-omics data analysis in multi-center studies with a fully open-source analytic platform
Source: PLoS Comput Biol. 2024 Dec 9;20(12):e1012626. doi: 10.1371/journal.pcbi.1012626 (PMC11658699; doi:10.1371/journal.pcbi.1012626)
Supplement: Attachment — Submitted filename: response_reviewers.pdf [file pcbi.1012626.s001.pdf]

Reviewer: 1

Further remarks: We have added a new section titled "Validation and Application of OmicSHIELD using Real-world Methylation Data" to the manuscript as a strategic move to address the reviewer's comments and further bolster the credibility and utility of OmicSHIELD. This section clearly demonstrates the platform's capability to handle real-world omic data securely and efficiently, offering practical evidence of its applicability in real-world research scenarios.

Comments to the Author

1.1

Comment: The proposed tool seems to support basic multi-omic analyses in federated settings. Recent advances in AI have led to numerous methods that leverage deep learning technology to improve the predictive outcomes in a variety of health applications (e.g., deep survival analysis, cancer subtyping). However, it is not clear whether the proposed tool can support these emerging predictive analyses.

Response: We agree eith the reviewer that AI is an important advanced for FA, however, our tool, OmicSHIELD, is designed to enable privacy-protected, federated omic data analysis, such as genome and epigenome-wide association studies, across multi-centre studies. It provides a secure environment for these analyses, meeting the demands of biomedical researchers while ensuring data privacy. Although acknowledging the potential of AI and deep learning in health applications, OmicSHIELD's current framework focuses on state-of-the-art association omic analyses (e.g, GWAS, EWAS, …) rather than using deep or machine learning for predictive purposes. Thus, OmicSHIELD prioritizes supporting specific, widely used analyses to facilitate collaboration and address the immediate needs of researchers. If the reviewer is interested in creating predictive models for different outcomes he/she can use other DataSHIELD packages such as dsSurvival, dsML or ask DataSHIELD developers to implement other methods.

Action: We have added this paragraph to the introduction (page 4):

“OmicSHIELD is designed to enhance biomedical research by facilitating a wide range of omic association analyses in federated settings, ensuring data privacy and compliance.”

1.2

Comment: Aspects regarding data partitioning across sites are not discussed with adequate details. In one use-case (PCA), the data are assumed to be horizontally partitioned. However, practical application settings may exhibit sites with vertically portioned data. Therefore, it is not clear, whether the proposed solution can be applied in those settings.

Response: OmicSHIELD is designed for horizontally partitioned data, ideal for federated analysis in multi-center omic studies where each site has data on different individuals but collects the same variables. We are aware that the point raised by the author is important and hence, we are working on it but it is currently out of the scope of the present work.

Action: We have added the following paragraph to the introduction (page 3):

"OmicSHIELD is designed to analyze horizontally partitioned data, with no current support for vertically partitioned data."

1.3

Comment: While OmicSHIELD enhances the privacy protection of federated analysis with the combination of multiple privacy-protecting technologies (e.g., differential privacy), the authors provided limited information on the use of such techniques in practice. As a result, users are left alone in finding the right balance between privacy and usability. It would be beneficial to add a use-case demonstrating these privacy techniques, including their impact on the usefulness and privacy, which may provide users with useful guidelines.

Response: Given your comments and the comments from the other reviewers, we included in the manuscript further information on how privacy settings affect the usability of the results. We hope that with the provided information the users will be properly informed or at least oriented about that topic and how to leverage it.

Action: We have added a privacy configuration comparison under Use case 1 (page 12):

"After demonstrating the efficacy of OmicSHIELD in replicating the significance of p-values and identifying top hits, we further investigate the influence of differential privacy parameters on the usability and interpretability of our results. Differential privacy employs a privacy parameter, epsilon (ε), to balance data utility against privacy. A smaller ε value indicates higher privacy but potentially less accurate results, while a larger ε allows for greater accuracy at the cost of privacy. To quantitatively assess this trade-off, we conducted additional analyses varying ε values at 0.01, 0.05, 0.07, 0.09, 0.1 and 1. These experiments aim to portray how changes in ε affect the overall utility of our federated analysis results, particularly in terms of statistical power and the ability to detect significant associations. The outcomes of these analyses are depicted in the Figure 5.

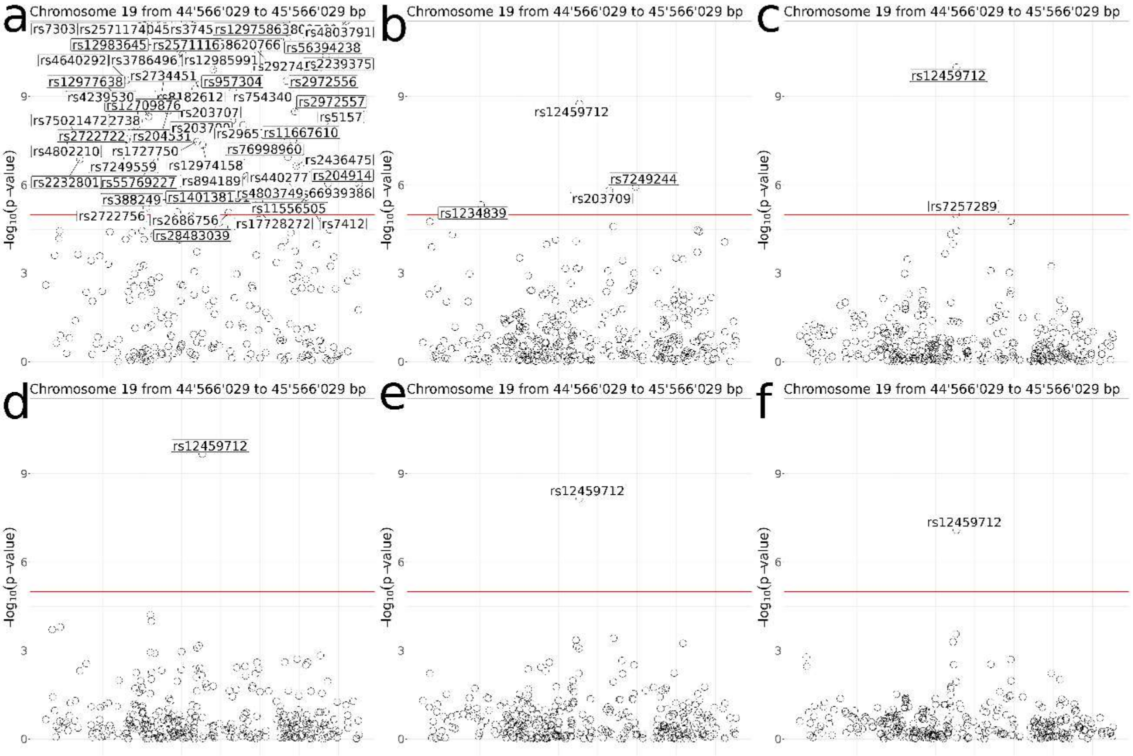

Figure 5. Impact of Differential Privacy Parameter (Epsilon) on the Usability of Federated Analysis Results. This figure presents the analysis of the effect of varying epsilon (ε) values on the detection of significant genetic associations in a federated omic data setting. Epsilon values tested include 0.01 (a), 0.05 (b), 0.07 (c), 0.09 (d), 0.1 (e) and 1 (f).

We observed a notable threshold at ε=0.09, which emerges as a critical point delineating the trade-off between maintaining privacy and achieving reliable statistical results. Below this ε value, our analysis revealed an increased incidence of false significant hits. Conversely, when ε values are set above this threshold, the stability and reliability of the results significantly improve. This stability indicates that ε=0.09 represents an optimal balance according to the authors findings. These findings underscore the importance of carefully selecting differential privacy parameters in federated omic data analyses to optimize both privacy protection and data utility."

1.4

Comment: Minor comments: There are some missing spaces in some sentences (e.g., abstract and introduction). Also, it seems some citations to DataSHIELD are missing (e.g., introduction).

Response: Thank you for the comment, we fixed the mentioned minor comments.

Reviewer: 2

Further remarks: We have added a new section titled "Validation and Application of OmicSHIELD using Real-world Methylation Data" to the manuscript as a strategic move to address the reviewer's comments and further bolster the credibility and utility of OmicSHIELD. This section clearly demonstrates the platform's capability to handle real-world omic data securely and efficiently, offering practical evidence of its applicability in real-world research scenarios.

Comments to the Author

2.1

Comment: Clarify the adversarial model and assumptions of the model with limitations of how privacy can be compromised, e.g., how does collusions between clients and data processors impact privacy? What can malicious adversaries do to breach privacy?

Response: In addressing the concern raised about the adversarial model and the assumptions regarding privacy protection within OmicSHIELD, we appreciate the opportunity to clarify our stance on these critical aspects. Our primary focus is on mitigating threats posed by insiders with authorized access but malicious intent. This model is particularly relevant in the context of federated data analysis, where multiple stakeholders, including data custodians and researchers, have varying degrees of access to sensitive data. Our adversarial model assumes that insiders, despite having legitimate access to the system, may attempt to utilize their position to infer individual-level information from the aggregated or analyzed data. In the context of data governance - if you give authorisation to data to a malicious actor, they will misuse the data. This is why the first mitigation is always strong data governance - the use of contractual obligations for the analyst. They would have to breach the legal contract to misuse the data / reidentify the data / output identifiable information. The OmicSHIELD software is adding an additional protective layer on top of the contractual governance to make it harder for misuse to occur. But the likelihood of a malicious actor being given authorization to do analysis on these data is extremely small - given the due diligence of researchers, their projects, etc for a formal data access requests and the legal obligation enforced by data access/collaboration contracts and employment contracts as well as termsa and conditions of data use.

Action: We have added a paragraph to the introduction clarifying the adversarial model (page 4):

"In federated data analysis of sensitive omic data, addressing data privacy threats is essential. Our adversarial model focuses on "insiders with authorized access but malicious intent" recognizing the significant risk from within, often overlooked. These insiders, including data custodians, researchers, and technical staff, might exploit their access to infer individual-level information from aggregated data, despite not having direct access to raw, unencrypted data. In the context of data governance, by giving authorization to data to a malicious actor, they will misuse the data. This is why the first mitigation is always strong data governance and the use of contractual obligations for the analyst."

2.2

Comment: Following up from the previous comment, is OmicSHIELD compliant with GDPR? This should be justified clearly.

Response: OmicSHIELD is designed to be GDPR-compliant because it uses privacy-enhancing technologies like differential privacy, returning aggregated data (not raw data) and filtering the minimum amount of data an operation is applied to (e.g. minimum individuals to compute a mean value) to safeguard data against inference attacks. Its operations adhere to GDPR principles of data minimization and purpose limitation, ensuring data stays within the owner's domain without cross-border transfers, thus maintaining data sovereignty and aligning with GDPR standards.

Both DataSHIELD and OmicSHIELD are already in use with real world data, the former being used for in excess of 10 years. In order to be deployed in use with real world data, data controllers will have conducted a risk assessment (IT security risk assessment and/or data protection impact assessment) defining the risks and mitigations for this federated analytic approach and satisfied their obligations within the context of GDPR as a data controller. It has recently been recomended that federated analysis that does not provide users access to identifiable personal data during analysis or outputs should not consider these users as controllers or processors under GDPR.

Bernier, Alexander, et al. "Reconciling the biomedical data commons and the GDPR: three lessons from the EUCAN ELSI collaboratory." European Journal of Human Genetics 32.1 (2024): 69-76.

Action: We have added a paragraph to the introduction (page 4):

"By enabling data to stay within its original repository and minimizing data movement, OmicSHIELD adheres to GDPR principles of data minimization and purpose limitation. Furthermore, OmicSHIELD's user-based architecture allows for audit trails and transparency, ensuring data usage is compliant with GDPR's accountability requirements."

2.3

Comment: How does OmicSHIELD compare to similar existing platforms in regards to adversarial assumptions and functionalities?

Response: We acknowledge that a comparative analysis with existing platforms could provide valuable insights into OmicSHIELD's unique contributions and its approach to addressing potential adversarial threats. Additionally the only similar tool, to our knowledge, is FAHME[1] that cannot be used since their tool is not open source, we cannot compare and we did not want to add comparisons on our manuscript.

[1]: Froelicher, David, et al. "Truly privacy-preserving federated analytics for precision medicine with multiparty homomorphic encryption." Nature communications 12.1 (2021): 5910.

2.4

Comment: I am not exactly sure about this statement: "However, pooled analysis in a multi-cohort setting is not recommended when data are heterogeneous among cohorts". Does this mean mega-analysis is not recommended when there is heterogeneity among populations? Can authors provide an example for a mega-analysis would fail in such as case?

Response: Mega-analysis emphasize uniform methodology across different centers, aiming for consistency in execution. However, this approach often overlooks regional variations such as differences in personnel quality, subject demographics, and prognosis. These variations, while

hidden within mega-analysis, become more transparent through a meta-analysis approach. Moreover, performing mega-analysis on heterogeneous data, would require the use of mixed-models, which are more computationally expensive and would require implementing scalable methods for that purpose as we did for glm's which is not trivial, we based our implementation on that reference.

Action: We have added a reference (page 3) to justify our sentence:

Shrier, Ian, Robert W. Platt, and Russell J. Steele. "Mega-trials vs. meta-analysis: precision vs. heterogeneity?." Contemporary clinical trials 28.3 (2007): 324-328.

2.5

Comment: In Figure 3, what are the non-disclosive statistics? Unless these are encrypted, it is not clear how a statistic can be non-disclosive.

Response: The non-disclosive statistics are aggregated values, e.g. mean values, beta values, p-values, etc. They are described as non-disclosive statistics as aside from being aggregated values, they pass through a series of filters described in the "Security" section so that they do not disclose personal information from the data that allows for re-identification of individuals.

2.6

Comment: It is very hard to follow the formulations without description of the symbols (e.g. page 18) and lack of appropriate mathematical notations which are currently rendered as text.

Response: We thank the reviewer for carefully reading and checking the manuscript. Related to this point, we would like to clarify that the issue you encountered appears to be related to the limitations of the journal's software in rendering mathematical notations and symbols from our original Word document submission. In our submission, we ensured that all mathematical formulations were accompanied by detailed descriptions of the symbols used and that we employed appropriate mathematical notation for clarity and accuracy. We have reviewed our original submission to verify that all mathematical notations and descriptions of symbols are correctly presented and comprehensively explained. We hope the reviewer can read all the details in the current version.

2.7

Comment: In figure 4, it is mentioned in one place that epsilon=3 is used for protection. This privacy budget is not justified since epsilon=3 is a fairly relaxed privacy budget. It is necessary to decrease the budget and compare results.

Response: We acknowledge the concern that an ε value of 3 might be perceived as a relaxed privacy budget, potentially compromising the stringent privacy standards required for sensitive omic data. In response to the reviewer's comment, we conducted a comprehensive sensitivity analysis to explore the effects of varying ε values on the usability and interpretability of our results, specifically focusing on ε values lower than previously used.

Action: We have added a privacy configuration comparison under Use case 1 (page 12):

Please refer to response 1.3 to read it.

2.8

Comment: Following from previous comment, it is not clear how DP is implemented into OmicSHIELD. I think OmicSHIELD uses DataSHIELD's functions to impose DP but it is necessary to clarify what type of DP is implemented into OmicSHIELD with all fine grained details so that users can understand how privacy is preserved. Generally, DP-based methods do not perform well on large dimensional datasets.

Response: This comment is related to different ones. Please take a look at responses 1.3, 2.9, 2.10 where we believe this comment has been answered. We believe those responses shed light on how DP is implemented on our solution.

2.9

Comment: Following from previous comment, \Delta f is estimated via simulations (Page 18), which means it is not theoreticaly derived and thus it does not lead to exact DP.

Response: As pointed out on the “Differential privacy” section of the manuscript, we estimate \Delta f (in some cases) using a re-sampling method described on [2]. We agree it does not lead to exact DP but we are confident on the method and the results described by their authors.

[2]: Rubinstein, Benjamin IP, and Francesco Aldà. "Pain-free random differential privacy with sensitivity sampling." International Conference on Machine Learning. PMLR, 2017.

2.10

Comment: Following from previous comment, \Delta f calculation should be clarified for all data that are exchanged in all protocols.

Response: We thank the reviewer for pointing out this important issue. In our implementation, we employ a resampling method to estimate \Delta f across all differential privacy transactions.

Action: We have added a sentence on the differential privacy section making that clearer (page 19):

“This resampling method has been used on all the analysis methods that return results to the client”.

2.11

Comment: Figure 5 shows a map of how data is distributed for expression/methylation analysis. Does that mean OmicSHIELD was run on multiple centers to perform a pooled analysis?

Response: The map portrays the organization of the project itself and how data on the project is analyzed through DataSHIELD. For reproducibility purposes of the use case, we have also placed the different datasets together on the same server (to ensure that it is available). When performing multiple connections to the same server, we mimic the same behavior as one dataset-one

connection, which is the same as pointing every connection to a different server. Therefore, using just one server, we perform a pooled analysis in the same way as it would be done across a distributed set of servers.

Action: We have added a clarification to the manuscript (page 15):

"For the sake of reproducibility, we included all the data on the same server, then different connections to it are performed, this replicates the same situation as having different servers scattered across different countries."

2.12

Comment: The privacy budget for DP is not reported for expression/methylation use case. Authors should show how the budget changes the accuracy of the final results.

Response: We have chosen to use the GWAS use case to provide a clear and concise illustration of how differential privacy epsilon value affects the usability of the outcome. Including a comprehensive analysis of the DP budget across multiple use cases would significantly increase the manuscript's length and dilute the focus on OmicSHIELD's novel contributions to federated privacy-protected omic data analysis.

Action: We have added the report of privacy budget for DP on the expression/methylation use case (page 15):

"The differential privacy settings used on this use case is ε=3."

Please refer to response 1.3 to read the analysis of DP budget.

2.13

Comment: There were no use cases for PCA, EWAS, and PRS.

Response: The use case for EWAS was already present in the manuscript (see pages 13-15). Regarding the inclusion of use cases for PCA and PRS, we aimed to balance the manuscript's comprehensiveness with its readability and length. We selected use cases that best demonstrate the software's application in real-world research scenarios, emphasizing its potential to facilitate collaborative studies while ensuring data privacy. Further examples are available on the online bookdown we have published along this manuscript.

Action: We have added the following sentence to point it out to the readers (page 4):

"On it, readers will find further use cases than the ones described on this manuscript."

2.14

Comment: In method descriptions, the "Data Processors" and "Clients" seems to be different entities but they were not clearly mentioned in the main text. I think DP_i refers to a data processor at the local site where data is stored. The setup of analysis should be clarified at the beginning of the study.

Response: We thank the reviewer again for carefully reading the text and pointing out this issue. We have corrected this misunderstanding.

Action: We have added a paragraph on the methods section (page 18):

"Along this section we describe some of the algorithms, there are two entities mentioned; data processors (DPs) and the client. The DPs are the servers where the data is stored and the analysis is performed. The client, typically represented by the researcher, sends instructions to the DPs for data analysis and receives back the results"

2.15

Comment: The details of PCA algorithm is not clear about how and why it would provide the same results as a pooled PCA analysis. DP_i sends u_i d_i to the client and client merges these and performs a final PCA. Do we expect that this will be the same as PCA on pooled data? Please provide more justification for this algorithm.

Response: We agree with the reviewer that not proving enough details can be a problem, but this is beyond the scope However, to facilitate that people understand the method we have provided another reference so readers can understand the method

Action: We have changed the reference to one that clearly explains the method and how it works (page 28).

Iwen, Mark A., and B. W. Ong. "A distributed and incremental SVD algorithm for agglomerative data analysis on large networks." SIAM Journal on Matrix Analysis and Applications 37.4 (2016): 1699-1718.

2.16

Comment: In PCA algorithm, sites need access to p_j, which must also be calculated in a privacy-preserving manner. It is not clear how this is accomplished.

Response: p_j stands for the probability of MAF that is computed as the mean of the vector of genotypes which are encoded as 0,1,2. Therefore, computing the mean is privacy preserving since it is an aggregated value.

Action: We have added an "Allele frequency" paragraph on "Methods" describing it (page 19):

"To compute the allele frequency using horizontally partitioned data, we use the following algorithm, where the multi-layered security approach of OmicSHIELD can be appreciated, precisely on steps 1.2, to 1.4.

DP_i: Data processor (Server node)
geno: Encoded genotype data

Step 1: Intermediate allel frequency
  1: Each DP_i computes allele_frequency(geno) [MAF_i, n_i]

ISGlobal Barcelona Institute for Global Health
info@isglobal.com
www.isglobal.org
Campus Clínic
Rosselló, 132
08036 Barcelona, Spain
T +34 93 227 1806
Campus Mar
Doctor Aiguader, 88
08003 Barcelona, Spain
T +34 93 214 7300

2: Each DP_i computes differential privacy \delta f
3: Each DP_i applies diff_privacy(MAF_i) [MAF_i]
4: Each DP_i checks MAF_filter(MAF_i) [MAF_i]
5: Each DP_i returns MAF_i to the client
Step 2: Client aggregation
1: The client receives MAF_i and n_i
2: The client sums MAF_i and divides by the sum of n_i
3: The client returns the pooled MAF results [final_results]"

2.17

Comment: Figure 6: What was the federated settings used in this plot?

Response: Thanks for pointing that out. This plot was computed without using differential privacy.

Action: We have added the information to the manuscript (page 21):

"When computing this plot, DP was turned off."

2.18

Comment: Following up from previous comment, the authors should provide the time/network usage with a realistic analysis scenario. Currently I could not find any reporting of network requirements of the methods.

Response: The manuscript already incorporates Plot 6, demonstrating the time efficiency of OmicSHIELD for real-world applications, indicating that further detailing of time usage is not needed. OmicSHIELD communicates by only transmitting concise results tables from servers to client (unlike other methods such as homomorphic encryption), thus eliminating the need for extensive data transfer. This approach lowers network demands, which we believe makes reporting on network usage unnecessary for our solution.

2.19

Comment: For PRS analysis authors state: "For this reason, the client does not receive any results when performing a PRS, results are instead stored on local study servers to be used as covariates in a GWAS or other aggregating functions."

Please provide some examples of how PRS estimates can be used as covariates in a GWAS or other aggregating functions.

Response: In OmicSHIELD, PRS are calculated locally at each study server to protect data privacy. These PRS estimates are then used as covariates in further analyses to adjust for genetic predisposition or to study how genetic risk interacts with other factors. PRS are also applied in GLMs to examine the relationship between genetic risk and phenotypes or other covariates. For example, a GLM might assess how genetic predisposition, as indicated by PRS, interacts with environmental or lifestyle factors in affecting disease risk, offering insights into gene-environment interactions and identifying population subgroups at increased risk.

Action: Modified the statement slightly and added a reference where they use the PRS results as a variable on a GLM (page 23):

“results are instead stored on local study servers, where they can be employed as covariates in subsequent analyses such as GWAS to control for genetic predisposition when identifying novel genetic associations, or utilized within GLM to explore interactions between genetic risk and environmental or lifestyle factors[3].”

[3]: Rodrigue, Amanda L., et al. "Specificity of psychiatric polygenic risk scores and their effects on associated risk phenotypes." Biological Psychiatry Global Open Science 3.3 (2023): 519-529.

Reviewer: 3

Further remarks: We have added a new section titled "Validation and Application of OmicSHIELD using Real-world Methylation Data" to the manuscript as a strategic move to address the reviewer's comments and further bolster the credibility and utility of OmicSHIELD. This section clearly demonstrates the platform's capability to handle real-world omic data securely and efficiently, offering practical evidence of its applicability in real-world research scenarios.

3.1

Comments to the Author

Comment: The paper introduces an open-source tool which is very interesting and useful for the community. Yet, the methods described in the paper lack technical novelty.

Response: We value your feedback regarding the technical innovation of OmicSHIELD. Our aim was to fill a crucial void in federated omic data analysis tools, focusing on privacy protection and accommodating various omic data types securely across multiple centers. Although some techniques like differential privacy and federated learning are not new, our innovation is in combining these into an open-source framework tailored for omic data that solves real-world data demands.

3.2

Comment: The paper itself, including the title, claim the system to be secure and private. Security and privacy are two different concepts that should be made clear in the respective subsections. Federated learning/analysis itself is not privacy-preserving due to several attacks. The authors state the system is privacy-preserving via its disclosure traps, control of output, and differential privacy. Also, authors state "the activity of authorized researchers can be audited and checks made to assess whether inference attacks have been performed" but there is no detailed information about how these audits are made. Eliminating inference attacks through only auditins is very challenging.
Without further details, it is very hard to quantify the privacy that the system provides (as also stated by the authors in Discussion with "but to personally assess and control the disclosure-prevention and differential-privacy mechanisms." If personal assesment is needed, this already underlines the system is not privacy-preserving by default. In the methods authors also state "Moreover, differential privacy can be applied to the results that the client receives."-> this should be mandatory for a system with aforementioned claims. I suggest authors to give a detailed explanation on all these issues.

Response: Thank you for such an insightful comment. Given the corrections and clarifications we have performed to address all the comments raised by you and the other reviewers, we believe that the concerns you express here have been properly addressed. Please take a look at responses 1.3, 2.1, 2.5, 2.9, 2.10 and 2.16; we hope they will provide the required details.

3.3

Comment: Regarding my previous comment, there is no experiment with differential privacy and different epsilon values. To protect privacy with differential privacy, the epsilon values should be reasonable and experimenting with different epsilon values would help the reader to understand the utility loss and the cutoff points.

Response: To address this, we have conducted additional experiments within the first use case focusing on differential privacy. These experiments explore a range of ε values to assess their impact on the utility loss and identify practical cutoff points that balance privacy protection with the usability of analysis results. This study aims to guide researchers in selecting appropriate ε values for their studies, offering a clearer understanding of the privacy-utility trade-off inherent in differential privacy.

Action: Added an experiment to explain the effect of epsilon on the results. Please refer to response 1.3 to read the analysis of DP budget.

3.4

Comment: In introduction, authors state “To address this important issue, different privacy-protecting techniques such as federated learning (FL), differential privacy (DP), homomorphic encryption (HE), and secure multi-party computation (SMPC) have been developed, some or all of which may be adopted” -> federated learning alone is not a privacy-protecting technique as shown by most attack works. It should always be combined with a ‘privacy-preserving’ mechanism. Also, authors should add the relevant citations (works use DP,SMC, or HE) in this sentence.

Response: We acknowledge that federated learning, in its basic form, does not inherently protect privacy and is susceptible to various inference attacks. Therefore, we agree that it is more precise to describe federated learning as a framework that facilitates the analysis of decentralized datasets, which, when combined with privacy-preserving mechanisms such as Differential Privacy (DP), Secure Multi-party Computation (SMPC), or Homomorphic Encryption (HE) offers privacy protection of the data being analyzed.

Action: We have modified the manuscript and added the correspondent references (page 3):

“To address this important issue, different privacy-protecting techniques such as federated learning (FL) in combination with differential privacy (DP)[4], homomorphic encryption (HE)[5], and secure multi-party computation (SMPC)[6] have been developed, some or all of which may be adopted [7]”

[4]: Dwork, Cynthia. "Differential privacy." International colloquium on automata, languages, and programming. Berlin, Heidelberg: Springer Berlin Heidelberg, 2006.
[5]: Mohialden, Yasmin Makki, et al. "Secure Federated Learning with a Homomorphic Encryption Model." International Journal Papier Advance and Scientific Review 4.3 (2023): 1-7.
[6]: Yan, Yang, et al. "Multi-participant vertical federated learning based time series prediction." Proceedings of the 8th International Conference on Computing and Artificial Intelligence. 2022.
[7]: He, Zaobo, et al. "Inference attacks and controls on genotypes and phenotypes for individual genomic data." IEEE/ACM transactions on computational biology and bioinformatics 17.3 (2018): 930-937.

3.5

Comment: Algorithms developed by researchers could potentially be used alongside genotype-phenotype associations from genetic association studies by an attacker to predict genotypes and phenotypes of target individuals based on genome information shared by individuals or their relatives [11]. -> more relevant citation is needed for this claim such as the reconstuction attacks or kinship attack papers.

Response: We also agree with the reviewer that further references should be added to justify this important point.

Action: We have added these new references (page 3):

Ayoz, Kerem, et al. "The effect of kinship in re-identification attacks against genomic data sharing beacons." Bioinformatics 36.Supplement_2 (2020): i903-i910.

Ayoz, Kerem, Erman Ayday, and A. Ercument Cicek. "Genome reconstruction attacks against genomic data-sharing beacons." Proceedings on Privacy Enhancing Technologies. Privacy Enhancing Technologies Symposium. Vol. 2021. No. 3. NIH Public Access, 2021.

3.6

Comment: "This approach has been adopted as a countermeasure to inference attacks using complex queries [23]:" -> the authors should provide a proof for this claim and perform known attacks.

Response: Performing and reporting results of different attacks was left out of the scope of our manuscript; nevertheless, we have added further cites of how differential privacy protects from inference attacks where the authors have already demonstrated that this approach can be used to prevent attacks

Action: We have added two references:

Ye, Dayong, et al. "One parameter defense—defending against data inference attacks via differential privacy." IEEE Transactions on Information Forensics and Security 17 (2022): 1466-1480.

Chen, Junjie, Wendy Hui Wang, and Xinghua Shi. "Differential privacy protection against membership inference attack on machine learning for genomic data." BIOCOMPUTING 2021: Proceedings of the Pacific Symposium. 2020.

3.7

Comment: "the differential privacy mechanisms we implement are intended to cause additional difficulty for such attacks." -> additional difficulty does not make a system secure and private as implied by the title.

Response: We agree with the reviewer, but with the sentence we want to say that we can't never have full control on the attacks, as portrayed by reference [23], inference attacks on MAF calculations (protected by differential privacy) are still prone to disclosing information. Our solution implements differential privacy + MAF filtering as a layered solution, therefore against that attack we reference, the differential privacy is a layer and not a complete privacy guarantee, which can be accomplished when combined with the MAF filter we reference after that sentence.

[23]: Almadhoun, Nour, Erman Ayday, and Özgür Ulusoy. "Inference attacks against differentially private query results from genomic datasets including dependent tuples." Bioinformatics 36.Supplement_1 (2020): i136-i145.

3.8

Comment: In Section “Omic analytic capabilities” authors state “OmicSHIELD contains functionalities to perform three types of omic data analysis: GWAS, DGE and EWAS” -> It is the first time DGE appears and authors should explain this acronym for the readers that are not familiar with the terminology

Action: We thank the reviewer for finding this missing information. We have included what the acronym stands for in the manuscript (Differential Gene Expression)

3.9

Comment: There are other relevant works that authors should discuss.

Response: We thank you the reviewer for pointing out these works, however we feel the proposed works are indeed related to federated learning or about differential privacy, but mainly they are pretty much unrelated to the work we present and the goals our solution try to meet. For that reason, we did not include further discussion about them as we believe that it will not add any valuable context to our manuscript.

3.10

Comment: Minor comments; typos/spaces/etc.

Response: Thank you for the comment, we fixed the minor issues.
