# Supplementary material for: Federated privacy-protected meta- and mega-omics data analysis in multi-center studies with a fully open-source analytic platform
Source: PLoS Comput Biol. 2024 Dec 9;20(12):e1012626. doi: 10.1371/journal.pcbi.1012626 (PMC11658699; doi:10.1371/journal.pcbi.1012626)
Supplement: Attachment — Submitted filename: cover_letter_and_response_reviewers.docx [file pcbi.1012626.s002.docx]

The PLOS Comp Biol Team

Carlyle House, Carlyle Road, Cambridge CB4 3DN | United Kingdom

ploscompbiol@plos.org | Phone +44 (0) 1223-442824

plos.org | ploscompbiol.org | @PLOSCompBiol

Barcelona 10^th^ June 2024

Dear Editor,

We would like to thank you and the reviewers for the time and effort spent evaluating our manuscript titled “Federated privacy-protected meta- and mega-omics data analysis in multi-center studies with a fully open source analytic platform” (Manuscript ID: PCOMPBIOL-D-23-01738R1). We appreciate the constructive feedback provided in this second review.

After carefully addressing all the reviewers’ comments and making substantial revisions to our manuscript, we believe it has been significantly improved. Therefore, we respectfully request that our revised manuscript be reconsidered for publication and subjected to a new review if necessary. Below is a summary of the key changes and improvements made in response to the reviewers’ feedback

Thank you for considering our request. We look forward to your favorable response and are available to provide any additional information or clarification required.

Sincerely,

Juan R Gonzalez

Corresponding author

## Reviewer 1

1.1

**Comment:** It is still not clear how the proposed privacy techniques work in synergy to protect privacy. For example, the allele frequency estimation algorithm shows how differential privacy and thresholding are used, but those techniques are not directly deployed in PCA and GWAS analysis. The proposed framework should support diverse analytics with a consistent privacy protection. If different privacy mechanisms are used for each analysis, authors should justify the choice of the mechanism.

**Response:** We thank the reviewer for pointing out his/her doubts regarding PCA and GWAS privacy. In the case of GWAS, we implemented differential privacy when writing the code of the algorithm, but it slipped through our mind mentioning it properly on the manuscript. Regarding the PCA, we suggested on the manuscript that it is possible to implement differential privacy to the block method. Following reviewer’s recommendations, we have implemented it in the new version.

**Action:** We have added a clear statement regarding the inclusion of differential privacy to GWAS on methods (page 21):

*“Even though is not explicitly mentioned on the pseudo-algorithm, differential privacy is also implemented on this algorithm and applied to all the operations that return aggregated results to the client. In this case it is trivial to see that there is no need to perform a sensitivity analysis, given the genetic data is encoded as [0, 1 ,2] integer values.”*

And we have also rephrased our explanation of differential privacy on the PCA on methods section (page 20) to include our new implementation:

*“Moreover, differential privacy is applied to the results that the client receives following the aforementioned methodology.”*

1.2

**Comment:** Figure 5 is very helpful in illustrating the impact of different values of the privacy parameter on the usability. Yet, it is not clear how the sensitivity analysis is performed. One concern is that the privacy protection illustrated may hold at variant-level, resulting in a patient-level differential privacy protection that is much lower (e.g., multiple variants are correlated). What is the overall privacy guarantee for each individual?

**Response:** Thank you for the comment. We agree that when multiple variants are correlated, the disclosure risk can be increased even though we are adding differential privacy to the algorithm. However, given our adversarial model (see response 1.4 for further information), we think this is out-of-scope of our manuscript , as DataSHIELD users are required to adhere to established data governance rules for data use. Our software enforces these rules, thereby mitigating further risks.

**Action:** We have added an additional paragraph expressing this on the methods (page 18):

*“In the case of GWAS, this method guarantees privacy at variant-level, the disclosure risk could be increased on the event that multiple variants are correlated; given the adversarial model of our software this case is out-of-scope of this research, as DataSHIELD users are required to adhere to established data governance rules for data use. Our software enforces these rules, thereby mitigating further risks.”*

1.3

**Comment:** Additional details regarding the computation of the sensitivity and noise perturbation would help the reader.

**Response:** We believe that on general terms, there is enough information about the algorithms we have used to compute the sensitivity and the noise perturbation. Nevertheless, to add more information we have detailed the resampling method we use to perform the sensitivity analysis.

**Action:** We have added the resampling method pseudo-algorithm on the methods (page 18):

*The resampling algorithm works as follows:*

*A: Function to which differential privacy is to be applied*

*D: Complete dataset*

*D1,2: Dataset missing a random individual*

*N: Number of resamples*

*Step 1: Derive D1,2 randomly from D*

*Step 2: Compute A(D1)*

*Step 3: Compute A(D2)*

*Step 4: Compute* $\Delta fN$ *=* $\left\| A\left( D_{1} \right)-A(D_{2}) \right\|$

*Step 5: Redo steps 1-4 N times*

*Step 6:* $\Delta fN$ *= max(*${\Delta f}_{1,2,\ldots, N}$*)*

1.4

**Comment:** In the revision authors state that the proposed solutions should protect from “insiders with authorized access but malicious intent”. Please, provide further clarification about the adversarial model (e.g., honest-but-curios vs Byzantine).

**Response:** Thank you for pointing this out, we have added a better description of the adversarial model.

**Action:** We have added a paragraph to the introduction (page 4):

*“More precisely, this falls into the honest-but-curious (HBC) adversary, which can be described as “participant who will attempt to learn all possible information from legitimately received messages”. The main threat being controlled is the ability of the adversary to re-indentify an individual within a dataset.” [31]*

*[31] A. Paverd, A. Martin, and I. Brown, “Modelling and Automatically Analysing Privacy Properties for Honest-but-Curious Adversaries”*

1.5

**Comment:** There are still some typos in the introduction.

**Response:** Thank you for pointing out this issue, we have gone over the introduction section to fix the typos.

## Reviewer 2

2.1

**Comment:** Authors added a citation for the sampling approach to clarify the comment on \Delta f: "The assessment of Δ can be particularly complex for certain functions (e.g. limma + voom), hence a sampling method [50] is used to assess Δ."

It would be very useful to the reader provide how this function is implemented step-by-step for the case of GWAS as an example.

**Response:** We agree that a more detailed view on how this is actually implemented would be very helpful for the reader.

**Action:** We have added the resampling method pseudo-algorithm on the methods (page 18):

*The resampling algorithm works as follows:*

*A: Function to which differential privacy is to be applied*

*D: Complete dataset*

*D1,2: Dataset missing a random individual*

*N: Number of resamples*

*Step 1: Derive D1,2 randomly from D*

*Step 2: Compute A(D1)*

*Step 3: Compute A(D2)*

*Step 4: Compute* $\Delta fN$ *=* $\left\| A\left( D_{1} \right)-A(D_{2}) \right\|$

*Step 5: Redo steps 1-4 N times*

*Step 6:* $\Delta fN$ *= max(*${\Delta f}_{1,2,\ldots, N}$*)*

## Reviewer 4

4.1

**Comment:** The privacy protection of the proposed software is mainly based on Differential Privacy. The client software may be used by a user who performs analyses frequently. Since the necessary privacy budget depends on the number of queries, it would be beneficial to present ideas on how to set the privacy budget and how to limit the number of queries in practice.

**Response:** We acknowledge the need to discuss ideas about how the number of queries should be limited since they can affect the privacy budget. Since it is not a topic that only affects our software, but rather the whole DataSHIELD federated analysis architecture, we added our concerns and future vision to the discussion section of the manuscript.

**Action:** We have added this paragraph to the discussion (page 17):

*“Given the proposed solution relies on differential privacy, it could be discussed that there has to be a system in place that limits the number of queries. That is indeed true, although it is not a mechanism that has to be included into OmicSHIELD, but rather a mechanism provided by the DataSHIELD architecture, given that all the different analysis package designed for it would benefit. As for the moment that this is not yet available, there is active logging of how the users interact with the federated platform, so an active control over the authorized users is possible to take into action.”*

4.2

**Comment:** The adversary model is described as malicious, but there is no explicit description in the manuscript why the omics data is protected from a malicious user. I think it may be better to describe why the system can protect data from malicious adversary. The security achieved by the system and potential security risks caused by major attacks are not clear.

**Response:** This comment is tightly related to comment 1.4 on this document. We have decided to further clarify the adversary model, and with that also included the main risk.

**Action:** We have added a paragraph to the introduction (page 4):

*“More precisely, this falls into the honest-but-curious (HBC) adversary, which can be described as “participant who will attempt to learn all possible information from legitimately received messages”. The main threat being controlled is the ability of the adversary to re-indentify an individual within a dataset.” [31]*

*[31] A. Paverd, A. Martin, and I. Brown, “Modelling and Automatically Analysing Privacy Properties for Honest-but-Curious Adversaries”*

4.3

**Comment:** The software is composed of various packages, but their roles in ensuring security are not clear. For example, a more detailed explanation of OPAL and DATASHIELD would be helpful for the reader to understand the proposed software.

**Response:** We agree that a more detailed explanation of Opal and DataSHIELD can be helpful. However, we feel that in the case of DataSHIELD we added enough references on the manuscript so that the reader can find plenty of details about it. We do not want our manuscript to be overloaded with details that distract the reader from the topic we want to discuss. In the case of Opal we agreed that there was not enough information, therefore we cited the paper where Opal was first described and introduced. Also, we reference to our bookdown where description, examples and how to manage data into this datawarehouse can be performed.

**Action:** We have added a reference and a sentence to introduce it on the introduction (page 3):

*“Two distinct data-warehouses exists for the FA DataSHIELD infrastructure, Opal and Armadillo that are elsewhere described [29]. A detailed bookdown can also be accessed to see examples of how Opal can be used to data management (*[*https://isglobal-brge.github.io/resource_bookdown/opal.html*](https://isglobal-brge.github.io/resource_bookdown/opal.html) *.”*

*[29] D. Doiron, Y. Marcon, I. Fortier, P. Burton, and V. Ferretti, “Software Application Profile Software Application Profile: Opal and Mica: open-source software solutions for epidemiological data management, harmonization and dissemination Denotes equal contribution”, doi: 10.1093/ije/dyx180.*
