# Supplementary material for: Federated privacy-protected meta- and mega-omics data analysis in multi-center studies with a fully open-source analytic platform
Source: PLoS Comput Biol. 2024 Dec 9;20(12):e1012626. doi: 10.1371/journal.pcbi.1012626 (PMC11658699; doi:10.1371/journal.pcbi.1012626)
Supplement: Attachment — Submitted filename: cover_letter_and_response_reviewers.docx [file pcbi.1012626.s003.docx]

The PLOS Comp Biol Team

Carlyle House, Carlyle Road, Cambridge CB4 3DN | United Kingdom

ploscompbiol@plos.org | Phone +44 (0) 1223-442824

plos.org | ploscompbiol.org | @PLOSCompBiol

Barcelona 1st August 2024

Dear Editor,

We would like to thank you and the reviewers for the time and effort spent re-evaluating our manuscript titled “Federated privacy-protected meta- and mega-omics data analysis in multi-center studies with a fully open source analytic platform” (Manuscript ID: PCOMPBIOL-D-23-01738R1). We appreciate the constructive feedback provided in this third review.

After carefully addressing all the reviewers’ comments and making substantial revisions to our manuscript by English native speakers who paid attention to typos and formatting issues, we believe it has been significantly improved. Therefore, we respectfully request that our revised manuscript be reconsidered for publication and subjected to a new review if necessary. Below is a summary of the key changes and improvements made in response to the reviewers’ feedback

Thank you for considering our request. We look forward to your favorable response and are available to provide any additional information or clarification required.

Sincerely,

Juan R Gonzalez

Corresponding author

## Reviewer 1

**Comment:** The inclusion of the pseudo-code for the resampling method originally proposed in [52] provides some useful information to the readers about the computation of the sensitivity. In my opinion, some improvement in the differential privacy section is needed. First, the approach in [52] provides an estimation of the sensitivity to achieve random differential privacy, which may result in a privacy protection that is weaker than the one provided by the traditional differential privacy model. Second, the number of rounds (N in the pseudo-code) cannot be arbitrary selected, but rather it needs to be carefully chosen to ensure that the privacy protection is met. The parameter N does not directly affect epsilon, instead a too small value may lead to failing the differential privacy guarantee overall. Note that [52] (Theorem 15) provides some lower bounds on the values of the parameters m and k in the original sensitivity sampler procedure, representing the number of samples and order statistics, respectively. Therefore, the statement “As the number of resamples performed can potentially impact differential privacy quality, we allow the data owner to configure this parameter”, may need to be adjusted to clarify the implications of these parameters. Also, pointers to the original paper indicating the privacy notion and information about the requirements on these parameters should be included.

**Response:** We thank the reviewer for this comment. We agree that the procedure used for the estimation of the sensitivity is for the achievement of random differential privacy and not the one provided by the traditional differential privacy model. We have clarified this in the section where the pseudo-code is presented.

**Action:** We replaced the statement and added a new reference (number 52) (see page 19)

**Old statement:** “As the number of resamples performed can potentially impact differential privacy quality, we allow the data owner to configure this parameter”

**New statement:** The used resampling method estimates the sensitivity to achieve random differential privacy [52]. The number of allowed resamples and the minimum values allowed for the other parameters of the method can be configured by the data custodian of each study while the default values are specified by the conditions defined in Theorem 15 of [51].”. We also cited the original paper about the requirements of these parameters.

[51]	B. I. P. Rubinstein and F. Aldà, “Pain-Free Random Differential Privacy with Sensitivity Sampling,” 2017.

[52]	R. Hall, L. Wasserman, and A. Rinaldo, “Random Differential Privacy,” Journal of Privacy and Confidentiality, vol. 4, no. 2, pp. 43–59, Mar. 2013, doi: 10.29012/jpc.v4i2.621.

**Comment:** There are still some typos and formatting issues. For example, the last three paragraphs in the introduction seem to be in a different font. Page 14: missing closing “)” after the link to Opal page. Page 21: “w’de added” -> “we have added”.

**Response:** We have gone through the entire manuscript and corrected all the typos we found (see version with track changes). This task has been done by three native English speakers: P.B, R.W and A.S.M. We also did some formatting changes in the manuscript and we can do any additional formatting is needed based on the Journal’s guidelines.

## Reviewer 4

**Comment:** The authors responded to most of my comments, and now I understand the adversarial model and the major risks they assumed.

Considering that the implemented privacy-preserving methods are not the type of methods that ensure no information is leaked (e.g., they guarantee only variant-level protection, have a query number limitation problem, and simple aggregation such as by disclosure traps cannot perfectly protect from re-identification attacks),

I think the manuscript includes rather over-advertisement descriptions such as:

- OmicSHIELD ensures that individual-level information remains confidential.

- It allows complex analyses across multiple datasets without compromising data security.

and it maybe better to mitigate the description such that the readers notice the limitations of the privacy-enhancing methods used in the proposed software.

**Response:** We thank the reviewer for this comment. We modified the statements that looked as over-advertisement descriptions.

**Actions:** The statements in page 2 have been modified as follows:

“OmicSHIELD ensures that individual-level information remains confidential”

has been replaced with

“OmicSHIELD aims to mitigate disclosure risks associated with analysis of omics data”.

“This makes it an invaluable tool for researchers looking to perform complex analyses across multiple datasets without compromising on data security. OmicSHIELD includes meta-level quality control checks and the ability to compute a wide range of analytical methods useful to obtain insights from omic data.”

has been replaced with

“This makes it a useful tool for researchers looking to perform complex analyses across multiple datasets. OmicSHIELD includes active disclosure control checks and the ability to compute a wide range of analytical methods useful to obtain insights from omic data.”
