# Supplementary material for: Federated privacy-protected meta- and mega-omics data analysis in multi-center studies with a fully open-source analytic platform
Source: PLoS Comput Biol. 2024 Dec 9;20(12):e1012626. doi: 10.1371/journal.pcbi.1012626 (PMC11658699; doi:10.1371/journal.pcbi.1012626)
Supplement: Attachment — Submitted filename: cover_letter_and_response_reviewer.docx [file pcbi.1012626.s004.docx]

The PLOS Comp Biol Team

Carlyle House, Carlyle Road, Cambridge CB4 3DN | United Kingdom

ploscompbiol@plos.org | Phone +44 (0) 1223-442824

plos.org | ploscompbiol.org | @PLOSCompBiol

Barcelona 4^th^ November 2024

Dear Editor,

We would like to extend our sincere gratitude to both you and the reviewers for your detailed evaluation and valuable feedback on our manuscript, titled “Federated Privacy-Protected Meta- and Mega-Omics Data Analysis in Multi-Center Studies with a Fully Open Source Analytic Platform” (Manuscript ID: PCOMPBIOL-D-23-01738R1). We appreciate the constructive guidance provided during the revision process, which has helped us enhance the clarity and robustness of our work.

In particular, we are grateful for the reviewer’s insightful comment regarding the implications of our use of random differential privacy (RDP). As suggested, we have now explicitly clarified in the manuscript that, while RDP offers certain computational benefits, it may lead to a weaker privacy guarantee compared to traditional differential privacy mechanisms. We agree that accurately selecting the number of rounds is crucial, as overly conservative values may cause computational inefficiencies, whereas excessively small values might compromise privacy guarantees.

To address this concern, we have introduced a new parameter (γ) to control the probability that the privacy loss could exceed ε, rather than leaving N as an adjustable setting for data managers. This approach ensures a balance between privacy and computational efficiency by automatically calculating the optimal N based on Theorem 15 in [51]. We have included detailed remarks reflecting these adjustments on page 18 of the manuscript. This response is further detailed below.

We thank you once again for the opportunity to refine our work through these valuable revisions. We believe these updates further strengthen our manuscript and enhance its relevance for readers. We look forward to your favorable consideration of our revised submission, and we remain available for any additional clarification or further revisions needed.

Sincerely,

Juan R Gonzalez

Corresponding author

## Reviewer 1

**Comment:** If you can make it clear in the manuscript that the used mechanism (random differential privacy) may result in a privacy protection that is weaker than the one provided by the traditional differential privacy model and also clarify the role of number of rounds in terms of how it needs to be carefully chosen and a very small value might fail the DP guarantees.

**Response:** We appreciate the reviewer's insightful comments regarding the importance of selecting an appropriate number of samples N in our sensitivity estimation procedure. We acknowledge that N is crucial for ensuring the differential privacy guarantees, as values that are too low may lead to inaccurate sensitivity estimates and potentially violate the desired privacy protection. Conversely, excessively high values of N can result in unnecessary computational overhead.

**Action:** To address this concern, instead of N being a configurable value by the data managers, we introduce a new parameter (γ) that measures the probability that the privacy loss can exceed ε. Then, following the lower bound provided in Theorem 15 on [51] we compute the N value that guarantees that. We have added the subsequent remarks that arise from these changes (on page 18):

“This method introduces an additional risk parameter to the differential privacy, γ, which represents the probability that the privacy loss can exceed ε. Following the conditions defined in Theorem 15 of [51] it is used to compute the number of resamples (N) to guarantee differential privacy under this assumption. A lower probability of privacy loss exceeding ε (low γ) will translate to a higher N. The privacy parameters can be configured by the data manager of each study while the default values are set to ε = 3 and γ = 0.1. This method implies that differential privacy is slightly weaker than traditional differential privacy (where sensitivity is assessed analytically) due to the probabilistic nature of its guarantee. However, by choosing a sufficiently small γ, the practical difference in privacy protection becomes negligible for most applications.”

This change is made in the DESCRIPTION file in the dsOmics package which is configurable by the data owner in the server side and makes reference to the differential privacy settings. Lines 83-85 in <https://github.com/isglobal-brge/dsOmics/blob/master/DESCRIPTION>

Options:

default.nfilter.diffP.epsilon=3,

default.nfilter.diffP.gamma=0.1,

We believe this addition enhances the clarity of our manuscript and provides readers with a better understanding of the privacy implications.

We also recognize the need for flexibility in real-world scenarios. Therefore, while we provide default parameter settings to ensure strong privacy guarantees, we allow data owners to specify lower values of γ if they want.
